# Hijacking of an autophagy-like process is critical for the life cycle of a DNA virus infecting oceanic algal blooms

**DOI:** 10.1111/nph.13008

**Published:** 2014-09-07

**Authors:** Daniella Schatz, Adva Shemi, Shilo Rosenwasser, Helena Sabanay, Sharon G Wolf, Shifra Ben-Dor, Assaf Vardi

**Affiliations:** 1 Department of Plant Sciences, Weizmann Institute of ScienceRehovot, 76100, Israel; 2 Department of Chemical Research Support, Weizmann Institute of ScienceRehovot, 76100, Israel; 3 Department of Biological Services, Weizmann Institute of ScienceRehovot, 76100, Israel

**Keywords:** algal blooms, Atg8, autophagy, coccolithophores, *Emiliani huxleyi*, *Emiliania huxleyi* virus, nucleocytoplasmic large DNA Virus

## Abstract

- Marine photosynthetic microorganisms are the basis of marine food webs and are responsible for nearly 50% of the global primary production. *Emiliania huxleyi* forms massive oceanic blooms that are routinely terminated by large double-stranded DNA coccolithoviruses. The cellular mechanisms that govern the replication cycle of these giant viruses are largely unknown.
- We used diverse techniques, including fluorescence microscopy, transmission electron microscopy, cryoelectron tomography, immunolabeling and biochemical methodologies to investigate the role of autophagy in host–virus interactions.
- Hallmarks of autophagy are induced during the lytic phase of *E. huxleyi* viral infection, concomitant with up-regulation of autophagy-related genes (ATG genes). Pretreatment of the infected cells with an autophagy inhibitor causes a major reduction in the production of extracellular viral particles, without reducing viral DNA replication within the cell. The host-encoded Atg8 protein was detected within purified virions, demonstrating the pivotal role of the autophagy-like process in viral assembly and egress.
- We show that autophagy, which is classically considered as a defense mechanism, is essential for viral propagation and for facilitating a high burst size. This cellular mechanism may have a major impact on the fate of the viral-infected blooms, and therefore on the cycling of nutrients within the marine ecosystem.

## Introduction

Phytoplankton are single-celled photoautotrophs that thrive in the upper illuminated layer of the oceans, form the basis of marine food webs and are responsible for nearly 50% of the global annual carbon (C)-based photosynthesis; hence they greatly influence global biogeochemical cycles (Field *et al*., 1998; Behrenfeld *et al*., 2006). Phytoplankton are the drivers of the ‘biologic pump’ where carbon dioxide is assimilated by photosynthesis to organic C, recycled in the top layer of the oceans or, ultimately, deposited on the ocean floor (Raven & Falkowski, 1999). Viral infections leading to cell lysis are estimated to induce the turnover of > 25% of the phytoplankton biomass, thus having a huge impact on ocean biogeochemical cycles by short-circuiting the flux of C and nutrients from phytoplankton and bacteria to higher trophic levels (Suttle, 2005; Bidle & Vardi, 2011).

Coccolithophores are among the most widespread classes of unicellular eukaryotic phytoplankton, and *Emiliania huxleyi* is the most abundant coccolithophore species in modern oceans (Winter *et al*., 1994). *E. huxleyi* forms massive annual blooms in temperate oceans and has a huge impact on biogeochemical cycles of C and sulfur, as well as on global climate regulation (Rost & Riebesell, 2004; Tyrrell & Merico, 2004). *E. huxleyi* blooms are reported to be routinely infected and terminated by a specific giant double-stranded DNA coccolithovirus, the *E. huxleyi* virus (EhV, *Phycodnaviridae*) (Bratbak *et al*., 1993; Brussaard *et al*., 1996; Wilson *et al*., 2002a). EhVs are large (*c*. 180 nm), have icosahedral symmetry, and are included in the nucleocytoplasmic large DNA virus (NCLDV) clade (Dunigan *et al*., 2006). With a genome size of *c*. 407 kb, EhV has unique genes, some of which have never been identified in other viruses (Wilson *et al*., 2005). Of major interest are genes encoding for an almost complete biosynthetic pathway for the production of sphingolipids that play a key role in the chemical arms race during this host–virus interaction (Wilson *et al*., 2005; Monier *et al*., 2009). Viral-derived sphingolipids were shown not only to mediate induction of host programmed cell death (PCD) but also to be major constituents of the viral lipidome, enriched in the virion membranes (Vardi *et al*., 2009). This viral-encoded biosynthetic pathway is functional during viral-induced bloom demise of natural coccolithophore populations (Pagarete *et al*., 2009; Vardi *et al*., 2009, 2012). Interestingly, hallmarks of PCD are induced in the host cell following infection by EhV both in cultures and in natural populations (Bidle *et al*., 2007; Mackinder *et al*., 2009; Vardi *et al*., 2012). Despite its huge importance in global biogeochemical cycles, very little is known about the cellular, biochemical, and molecular processes that govern infection of *E. huxleyi* by EhV.

Eukaryotes have developed many cellular mechanisms to defend against environmental stress, including attack by pathogens. One such highly conserved mechanism is autophagy, which facilitates the degradation of damaged organelles and undesirable macromolecules via a lysosomal degradative pathway (see Mizushima, 2007; Avin-Wittenberg *et al*., 2012; Li & Vierstra, 2012 and references within). Hallmarks of active autophagy are the production of double membrane vesicles (DMVs) termed autophagosomes, lipidation of the Atg8 protein (LC3 in mammals) with phosphatidylethanolamine (PE) to produce Atg8-PE that is bound to the growing autophagosome, and, lastly, maturation of the autophagosome by fusion to the lysosome (vacuoles in plants) and degradation of the sequestered cargo (He & Klionsky, 2009). Upwards of 30 proteins have been implicated as participants in the autophagic machinery in yeast, plants, and mammals (Klionsky *et al*., 2010; Avin-Wittenberg *et al*., 2012); however, very little is known about autophagy in phytoplankton. It was only recently reported that green algae contain the core autophagic machinery (Perez-Perez *et al*., 2010; Jiang *et al*., 2012), but experimental evidence for the function and environmental relevance of this process is lacking.

The crosstalk between autophagy and viral infection can be manifested by formation of DMVs and the impact it has on membrane structure in the cell. Many RNA viruses use the autophagic membrane as a scaffold for RNA replication (Prentice *et al*., 2004; Jackson *et al*., 2005; Dreux *et al*., 2009; Maier & Britton, 2012). Autophagy can also serve as an antiviral host defense mechanism, by subjecting the infecting viruses to autophagic-lysosomal degradation (Liang *et al*., 1998; Orvedahl & Levine, 2009). Lastly, autophagy can induce a switch between stress acclimation and initiation of a cell death biochemical cascade following stress of eukaryotic cells (Yu *et al*., 2004; Codogno & Meijer, 2005; Zalckvar *et al*., 2010). This switch can act as a proviral or antiviral strategy. In plants, autophagy plays a critical role in the hypersensitive response to pathogens (Liu *et al*., 2005; Kabbage *et al*., 2013). On the other hand, viruses may use cell death as a means of release from the infected cell, as shown for vaccinia virus intracellular mature viruses and suggested for EhV (Best, 2008; Roberts & Smith, 2008; Bidle & Kwityn, 2012). Nonetheless, the interplay between autophagy and infection by giant viruses of the NCLDV clade is poorly understood.

Here we report a functional role for an autophagic-like process in *E. huxleyi* and demonstrate that it is an essential component of the EhV replication cycle. We show that the *E. huxleyi* genome contains homologous components of the core autophagic machinery that are up-regulated during viral infection, concomitant with an increase in acidic vesicles within the cells. This autophagy-like process is essential for viral release from the host cells. We show that the membranes produced by this process are essential for construction and propagation of the virions, enabling the large burst size observed for EhV.

## Materials and Methods

### Culture growth and viral infection dynamics

The noncalcifying *Emiliania huxleyi* strain CCMP2090 (*E. huxleyi*) was used for this study. Cells were cultured in K/2 medium (Keller *et al*., 1987) and incubated at 18°C with a 16:8 h, light:dark illumination cycle. A light intensity of 100 μmol photons m^−2^ s^−1^ was provided by cool white LED lights. All experiments were performed with exponential phase cultures (5 × 10^5^–10^6^ cells ml^−1^). The virus used for this study is the lytic *Emiliania huxleyi* virus EhV201 (Schroeder *et al*., 2002). In all infection experiments, *E. huxleyi* CCMP2090 was infected with 1:50 volumetric ratio of viral lysate to culture (multiplicity of infection (MOI) of *c*. 1:1 viral particles per cell). When indicated, rapamycin bafilomycin or wortmannin (Sigma-Aldrich) in dimethyl sulfoxide (DMSO) was added to cultures by ×1000 dilution to reach a final concentration of 10 μM, 50 nM or 1 μM, respectively (concentrations where the observed affects were most pronounced but that did not affect growth rate). An equal volume of DMSO was added to all control cultures. For all experiments, *P*-value was calculated using Student’s *t*-test.

### Enumeration of cell and virus abundance

Cells were counted using a Multisizer 4 Coulter counter (Beckman Coulter, Nyon, Switzerland). For counting the extracellular viruses by quantitative PCR (qPCR), 0.5 ml samples were filtered through a 0.45 μm Millex-HV filter (Millipore) and boiled for 20 min. One microliter of the viral lysate was taken for each reaction; each sample was analyzed in duplicate. EhV DNA was quantified using primers against the Major Capsid Protein (*mcp*) gene, mcp1Fw and mcp90Rv (for primer sequence, see Supporting Information, Table S1). For intracellular viral DNA quantification, 1 ml of cells were collected by centrifugation (8000 ***g***, 3 min, 4°C), washed twice in fresh media and the DNA was released from the cells using REDExtract-N-Amp Plant PCR kit (Sigma-Aldrich) according to the manufacturer’s instructions.

The extract was diluted ×100 in water and 1 μl was used for qPCR analysis with the *mcp* primers as described earlier. All reactions were carried out in duplicate. For all reactions, Platinum SYBER Green qPCR SuperMix-UDG with ROX (Invitrogen) was used as described by the manufacturer. Reactions were performed on StepOnePlus™ real-time PCR Systems (Applied Biosystems) as follows: 50°C for 2 min, 95°C for 2 min, 40 cycles of 95°C for 15 s, 60°C for 30 s. Results were calibrated against serial dilutions of EhV201 DNA at known concentrations, enabling exact enumeration of viral abundance.

For all experiments, the *P*-value was calculated using Student’s *t*-test.

### Isolation and concentration of virions

Three liters of viral lysate of *E. huxleyi* were concentrated on a 50 kDa Tangential Flow Filtration system (Millipore) and viruses were separated by an OptiPrep gradient (25–40%, according to Lawrence & Steward, 2010) and washed three times on a 50 kDa Amicon filter (Millipore). Concentrated viruses were suspended in 200 μl PBS.

### Transmission electron microscopy (TEM)

A 500 ml culture was collected (8000 ***g***, 10 min, 20°C), resuspended in fixation media (2% glutaraldehyde, 4% paraformaldehyde, 2% acrolein in artificial sea water (ASW)), and fixed for at least 24 h at 4°C. The cells were then washed in ASW and postfixed in 2% osmium tetroxide, 0.5% potassium dichromate and 0.5% potassium hexacyanoferrate in ASW for 1 h, at room temperature, washed again and stained en bloc with 2% aqueous uranyl acetate for 1 h followed by ethanol dehydration. Samples were infiltrated with increasing concentrations of Epon EMBED 812 (EMS, Hatfield, PA, USA) and polymerized at 60°C. Thin sections (*c*. 70 nm) obtained with an Ultracut UCT microtome (Leica Microsystems, Wetzlar, Germany) were poststained with 2% uranyl acetate and Reynold’s lead citrate and examined using an FEI Tecnai T12 TEM operating at 120 kV. Images were recorded on an FEI Eagle 2Kx2K CCD camera.

### Cryotransmission electron microscopy

Clean, concentrated virions were applied to Quantifoil 1/4 grids (Quantifoil Micro Tools, Jena, Germany), with the addition of 16 nm fiducial gold beads, and then blotted and plunged into liquid ethane using a Leica EM-GP plunger (Leica Microsystems). Frozen specimens were transferred to Gatan 914 or Gatan 626 cryo-holders, and maintained at temperatures below −176°C inside the microscope. Specimens were observed with an FEI Tecnai F-20 TEM (FEI Corp., Hillsboro, OR, USA) operating at 200 kV. Images were recorded on a Gatan US4000 CCD camera (Gatan Inc., Pleasanton, CA, USA). Bilayer thickness was measured with the iTEM program (Olympus Soft Imaging Solutions, Münster, Germany).

### Immuno-TEM on isolated virions

Immuno-TEM was carried out as described in Tokuyasu (1986) with minor alterations to the method. Virions were fixed in 0.5% glutaraldehyde in ASW for 2–3 h, and then washed three times using a 50 kDa Amicon filter. An equal volume of 10% gelatin was added and the samples were incubated at 37°C for 30 min and then transferred to ice for 30 min to solidify the gelatin. For further fixation, specimens were covered with 0.5% glutaraldehyde in ASW and incubated for 24 h at 4°C. The virions embedded in the gelatin were cryoprotected by infiltration with 2.3 M sucrose for 24 h at room temperature and frozen by plunging into liquid nitrogen. Ultrathin (*c*. 75 nm) frozen sections were then cut with a diamond knife at −120°C. Sections were transferred to formvar-coated 200 mesh nickel grids and treated with CM (Conditioning media, 0.5% BSA, 1% glycine, in PBS) for 5 min followed by 12 h incubation with anti-Atg8 antibody (Abcam ab4753 diluted 1:30 in CM; Abcam, Cambridge, UK) at 4°C. After extensive washing in 0.1% glycine in PBS, the primary antibody was detected with antirabbit 10 nm colloidal gold conjugate (1:20 in CM, EMS). Grids were then stained with 2% uranyl acetate in H_2_O for 10 min and embedded in 2% methyl cellulose/uranyl acetate. Images were acquired using an FEI Tecnai T12 TEM operating at 120 kV. Images were recorded on an FEI Eagle 2Kx2K CCD camera.

### Staining with *in vivo* fluorescent lysosomal markers

For Lysosensor and monodansylcadaverine (MDC) staining, *c*. 10^6^ cells were concentrated by centrifugation at 14 000 ***g*** for 3 min and resuspended in 100 μl Lysosensor Green DND-189 (Molecular Probes, Eugene, OR, USA) or MDC (Sigma Aldrich), both diluted to a final concentration of 1 μM in filtered sea water (FSW). After 10 (Lysosensor) or 30 (MDC) min of dark incubation, the cells were washed twice in FSW. Fluorescence image data were obtained by an Olympus FluoView FV1000 IX81 Spectral/SIM Scanner confocal laser-scanning microscope, using a 1.35 NA UPLSAPO 60 oil objective. Samples were excited at 440 nm and observed with emission at 502–545 nm (Lysosensor) or 460–560 nm (MDC). Chlorophyll autofluorescence images were obtained by excitation at 638 nm and emission at 655–755 nm. Quantification of fluorescent staining was performed using an Eclipse (iCyt) flow cytometer. Lysosensor and MDC stainings were measured in the green channel (emission: 525 nm) following excitation at 488 nm. At least 10 000 cells were examined for each measurement.

### Infectivity assay

Extracellular viruses were fixed with 0.5% glutaraldehyde, incubated at 4°C for 30 min, then plunged into liquid N_2_ and kept at −80°C until analysis. After thawing, a 2:75 ratio of fixed sample to SYBR Gold stain solution (Invitrogen) was incubated for 20 min at 80°C, and cooled down to room temperature. SYBR Gold was prepared by diluting it into filtered Tris-EDTA (TE, 1:10 000) as specified by the manufacturer. Flow cytometric analysis was performed on an Eclipse (iCyt) flow cytometer, with a 488 nm excitation laser and 525 nm emission to yield viral-like particle (VLP) counts. An equal number of VLPs was taken for each treatment to a plaque assay according to Schroeder *et al*. (2002) and Wilson *et al*. (2002b). Essentially, 50 ml of cells at 10^6^ cells ml^−1^ were concentrated (3000 ***g***, 3 min) to 900 μl. One hundred microliters of virus at a concentration of 10^4^ VLPs ml^−1^ were added to the cells. After 2 h incubation under normal growth conditions, the virus–host mixture was mixed with 3 ml of K/2 media containing 0.2% agarose and poured onto a K/2 media solidified by 1.5% agarose plate. At 72 hpi, the plates were scanned using a Typhoon 9410 Variable Mode Imager (GE Healthcare, Little Chalfont, UK) and plaques were counted manually. Three biological repeats were performed, each containing three technical repeats. Student’s *t*-test was used to calculate the *P*-value.

### Subcellular fractionation and solubilization of Atg8

Fractionation of soluble and membrane fractions were carried out as in Perez-Perez *et al*. (2010), with minor changes. Essentially, *E. huxleyi* whole-cell extracts were prepared from 500 ml cultures at *c*. 10^6^ cells ml^−1^ that were centrifuged (10 000 ***g*** for 15 min at 4°C) and plunged into liquid nitrogen. Cells were then resuspended in lysis buffer (150 mM NaCl, 1 M Tris pH = 8, 0.5 M EDTA) and lysed by sonication (5 × 5.5 s cycles). Samples were centrifuged (500 ***g*** for 5 min at 4°C) to remove cell debris. The supernatant was centrifuged at 15 000 ***g*** for 15 min at 4°C to generate the membrane fraction. For Atg8 solubilization, the membrane pellet fraction was resuspended in lysis buffer containing 1% deoxycholate and incubated on ice for 1 h. Samples were then centrifuged at 100 000 ***g*** for 2 h to separate soluble from insoluble proteins. The pellet containing the insoluble proteins was treated with phospholipase D by incubating the membrane fraction at 37°C for 1 h with lysis buffer containing 2 U μl^−1^ *Streptomyces chromofuscus* Phosphlipase D (Enzo Life Sciences, Farmingdale, NY, USA). Reactions were stopped by addition of sample buffer.

### Immunoblot assays

Whole-cell proteins were extracted by sonicating a pellet of 250 ml of cells resuspended in RIPA buffer (25 mM Tris pH = 7.6, 150 mM NaCl, 1% NP-40, 1% sodium deoxycholate, 0.1% sodium dodecyl sulfate) and centrifuging (500 ***g*** for 5 min at 4°C) to remove the cell debris. Virion proteins were extracted by adding an equal volume of RIPA buffer to concentrated virions and boiling the sample for 10 min. Proteins were separated on a 6 M urea sodium dodecyl sulfate polyacrylamide gel electrophoresis (SDS-PAGE) and blotted onto polyvinylidene difluoride (PVDF) membranes. Anti-Atg8 (raised against the yeast Atg8, Abcam, ab4753) and the secondary horseradish peroxidase-conjugated antirabbit antibody (Sigma-Aldrich) were diluted 1:4000 and 1:10 000, respectively, in Tris-buffered saline containing 0.1% Tween 20 and 5% milk powder. The ECL-Prime western blotting detection reagent (GE Healthcare) was used for detection. Note that the antibody cannot distinguish between the *E. huxleyi* Atg8a and Atg8b protein sequences.

### RNA isolation and RT-PCR analysis

RNA was isolated from 250 ml cultures at time points as indicated with the RNeasy Plant Mini kit (Qiagen) according to the manufacturer’s instructions, followed by DNAse treatment with Turbo DNAse (Ambion). Equal amounts of RNA were used for cDNA synthesis with the ThermoScript RT-PCR system (Invitrogen). For transcript abundance analysis, Platinum SYBR Green qPCR SuperMix-UDG with ROX (Invitrogen) was used as described by the manufacturer. A list of primers for the detection of transcripts of *atg8a*, *atg8b*, *vps34*, *atg5*, *atg7*, tubulin and the viral *mcp* and *spt* genes is given in Table S1. Reactions were performed on StepOnePlus real-time PCR Systems (Applied Biosystems) as follows: 50°C for 2 min, 95°C for 2 min, 40 cycles of 95°C for 15 s, 60°C for 30 s. Transcript abundance was calculated by normalizing the results to expression of tubulin in each sample and to the expression of the control (uninfected) sample at the same time point.

### Multiple alignment of Atg8

Multiple alignment was performed with ClustalW version 2.1 using the default parameters (Larkin *et al*., 2007). The sequences used for alignment are as follows: Ehux atg8a, *Emiliania huxleyi* Atg8a, BK008760; Ehux atg8b, *Emiliania huxleyi* Atg8b BK008760; Scer, *Saccharomyces cerevisiae* NP_009475.1; Crei, *Chlamydomonas reinhardtii* XP_001699190.1; Atal atg8c, *Arabidopsis thaliana*_c NP_176395.1; Ptri, *Phaeodactylum tricornitum* XP_002185239.1; Tpse, *Thalassiosira pseudonana* XP_002287173.1 corrected based on expressed sequence tags (ESTs), an additional two amino acids added at N-term, MT; Pinf, *Phytophthora infestans* XP_002906463.1; Hsap, *Homo sapiens* GABARAPL1 NP_113600.1; Hsap, *Homo sapiens* LC3a NP_115903.1. *atg*5, *atg*7 and *vps*34 sequences were submitted to GenBank Third Party Annotation (TPA), under accession nos. BK008763, BK008764 and BK008762 respectively.

## Results and Discussion

### Ultrastructure analysis reveals formation of DMVs during viral infection of *E. huxleyi*

**Figure 1:**
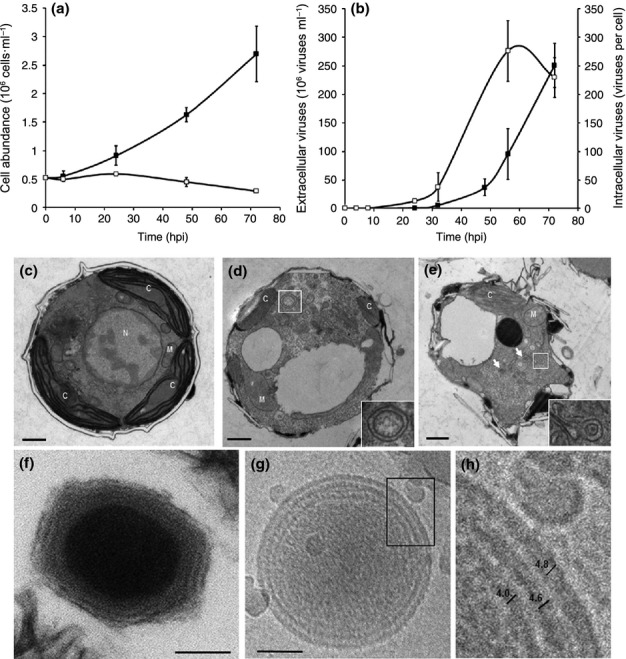
Infection dynamics and ultrastructure analyses of *Emiliania huxleyi* and its lytic virus EhV. (a) Assessment of host–virus interactions during the course of viral infection by quantifying cell abundance of infected (open squares) and uninfected (closed squares) cultures. (b) Intracellular (open squares) and extracellular (closed squares) viral counts of infected *E. huxleyi* cultures, as estimated by quantitative PCR (qPCR) using primers for the *mcp* gene (for a and b, *n* = 3; results presented are average ± SD.) (c) Transmission electron microscopy (TEM) analysis of a representative micrograph of *E. huxleyi* control cells. (d, e) Infected cells 24 h postinfection (hpi) by EhV. Degradation of mitochondria (M) and chloroplasts (C) is apparent. Arrows point to double membrane vesicles (DMVs). Bars, 500 nm. The insets in (d) and (e) show higher magnification of the boxed areas, depicting the DMVs. A larger version of (c–e) can be found in Fig. S5. (f) TEM analysis of a chemically fixed virion, the DNA is observed as the electron-dense material in the center of the virion. Three layers surrounding the DNA can be observed. (g) Cryotomography image of an isolated virion. (h) Higher magnification of the boxed areas are shown in (g), allowing measurement of the membrane-like structures. The numbers represent average lengths (nm) of 20 different measurements.

Infection of the noncalcifying *E. huxleyi* CCMP2090 (hereafter *E. huxleyi*) by the double-stranded DNA virus EhV201 revealed a lytic dynamic of infection whereby the host culture is lysed within 72 h postinfection (hpi; Fig. 1a). Intracellular viral DNA accumulated in the cells before the onset of release of extracellular viruses to the media (Fig. 1b). This host–virus temporal dynamics resembles the kinetics of infection observed in natural populations (Bratbak *et al*., 1993; Vardi *et al*., 2012). Transmission electron microscopy (TEM) of infected cells revealed compromised cells within 24 h of infection (Fig. 1c–e). Degradation of nuclear material is apparent, as well as shrinkage of the chloroplast. These observations are in agreement with previous data whereby a reduction in photosynthetic efficiency occurs after the onset of the lytic phase, concomitant to activation of PCD-like processes mediated by caspase activity (Bidle *et al*., 2007; Vardi *et al*., 2009; Kegel *et al*., 2010; Kimmance *et al*., 2014). Interestingly, in almost all the observed infected cells, we detected DMVs (Fig. 1d,e). Quantification of the DMVs in the TEM images of infected and control cells revealed a significant difference in their abundance. In infected cells, we counted 2.65 ± 0.24 DMVs per cell, whereas in control cells we observed 0.3 ± 0.1 DMVs per cell (average ± SE, *n* = 20, *P* < 0.05). The presence of DMVs indicates a major redistribution of cellular membranes during infection of *E. huxleyi* with EhV.

Transmission electron microscopy analysis of chemically fixed virions (Fig. 1f) suggested that the virion is composed of at least two, and possibly three, layers surrounding the electron-dense DNA core. We used cryotomography on unfixed virions to establish the characteristics of these layers (Fig. 1g,h). While we cannot unequivocally determine the nature of the three layers surrounding the DNA core, the thickness of the outer two layers, *c*. 4.6 and 4.8 nm, suggests that they are lipid bilayers (Hollinshead *et al*., 1999). These results are similar to those found in all the NCLDV-clade viruses, including two other members of the phycodnaviridae, PBCV1 and EsV1, that contain membranes internal to the capsid (Van Etten *et al*., 2002), and emphasize the substantial requirement for membranes during infection, the source of which is not known. The observed DMVs suggest the occurrence of membrane redistribution during infection, which may provide a source for the massive requirement of viral membranes.

### Infected cells exhibit hallmarks of an autophagy-like cellular process

**Figure 2:**
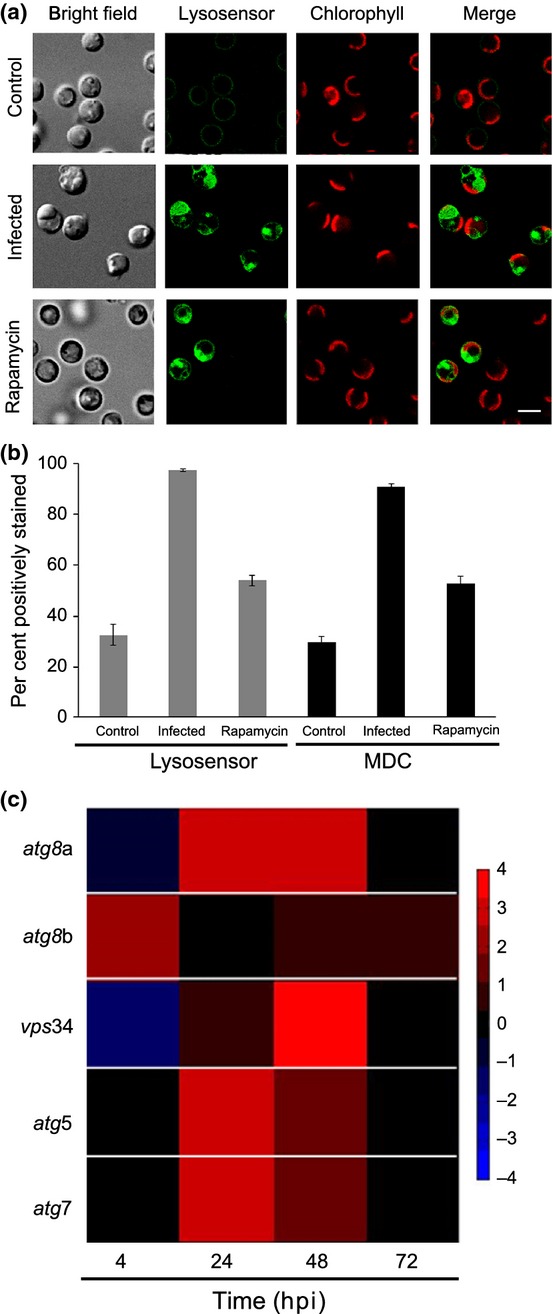
*Emiliania huxleyi* displays hallmarks of an autophagy-like process during infection. (a) Confocal micrographs of control, infected cells 24 h postinfection (hpi), or rapamycin-treated cells (positive control) that were stained with Lysosensor for detecting acidic compartments within the cells. Green, Lysosensor stain; red, Chl autofluorescence. Bar, 3 μm. See Fig. S6 for a green-magemta version of the same image. (b) Quantification of the fraction of monodansylcadaverine (MDC) or Lysosensor-positive cells by flow cytometer analysis of cultures stained with MDC or Lysosensor. Control, untreated; infected, cells infected with *Emiliania huxleyi* virus (EhV) at 24 hpi; rapamycin, cells treated with 10 μM rapamycin to induce autophagy. Results are an average of three experiments ± SD, 10 000 cells were counted in each. (c) Reverse transcription polymerase chain reaction (RT-PCR) analysis of selected homologs of core autophagy-related genes in cultures infected with the lytic EhV201 throughout the course of infection. Results are normalized to tubulin and to the uninfected control at the same time point. Data are presented as log_2_ fold change.

**Figure 3:**
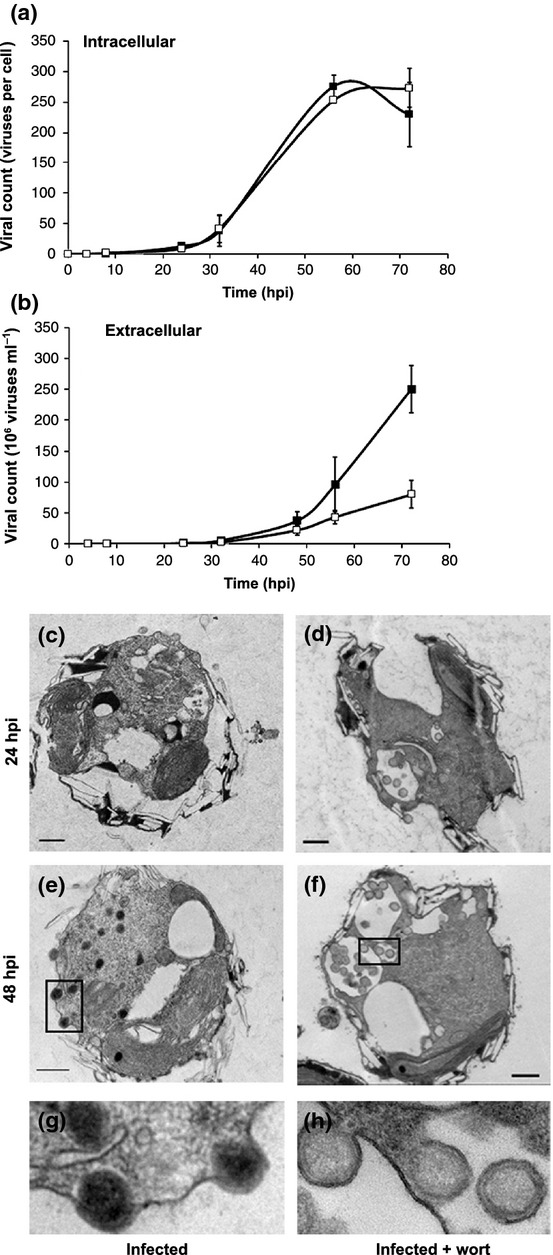
Inhibition of the autophagy-like process reduced viral release. Assessment of host–virus dynamics in response to wortmannin treatment by quantification of intracellular (representing viral DNA replication) (a) and extracellular (b) viral count during infection with (closed squares) and without (open squares) 1 μM wortmannin estimated by quantitative PCR (qPCR) using primers for the *mcp* gene (for a and b, *n* = 3; results presented are average ± SD). Transmission electron microscopy (TEM) analysis of infected cultures (c, e) and cultures infected in the presence of wortmannin (d, f) at 24 and 48 hpi. (g, h) Higher magnifications of the boxed areas in (e) and (f), respectively. Bars, 500 nm.

To study the major features of an autophagic-like process during infection of *E. huxleyi*, we stained infected cells with MDC and Lysosensor; both stain acidic compartments and are indicative of an active lysosomal degradative process within the cells. Indeed, by 24 hpi, 90% of the cells within the infected population were positively stained (Figs. 2a,b, S1). The profound effect of viral infection on induction of acidic compartments was even greater than that observed following application of the autophagy-inducer rapamycin as a positive control, emphasizing the major effect that infection has on the autophagic-like process within the host cells (Fig. 2a,b). In many host–pathogen systems, autophagic-lysosomal degradation serves as a cellular defense mechanism against viral infection, while many RNA viruses subvert the autophagic machinery to their advantage (Kirkegaard *et al*., 2004; Lin *et al*., 2010; Richards & Jackson, 2013). In our system, viral production is high despite the presence of lysosome-like compartments, suggesting that this induction is not used by the host as a defense mechanism and implying that EhV uses autophagy to benefit its replication cycle. Treatment of infected cells with bafilomycin, an inhibitor of lysosomal acidification, had no significant effect on viral production at 48 hpi (*P* = 0.7, Fig. S2). Interestingly, at 30 hpi, there was a significant elevation in extracellular viral abundance (*P* < 0.05), which raises the possibility that acidic lysosomal pH may delay viral release at the earlier stages of infection. However, we could not detect any viral particles within lysosomes by TEM analysis of infected cells, at any stage of infection (Figs 1d,e, 3c,e). This suggests that the EhV particles do not encounter the lysosome during regular infection, and may even block the fusion between the double-membrane autophagosome and the lysosome, at least at the later phases of infection.

### Homologs of core autophagy-related genes are up-regulated during viral lytic infection

Gene mining of the *E. huxleyi* genome (Read *et al*., 2013; Feldmesser *et al*., 2014) revealed homologs of the core autophagy-related genes; among them we identified the *atg*8 gene, which is central for autophagsome formation. *E. huxleyi* has two homologs of the highly conserved Atg8 (Atg8a and Atg8b, 13.4 and 15.9 kDa, respectively). Both homologs have the essential residues for activation and function, such as the glycines in position 120 and 126 and Phe77 and Phe79 (Fig. S3), all of which are essential for the cleavage at its C terminus before lipidation and downstream functions (Ichimura *et al*., 2000; Amar *et al*., 2006). We used RT-PCR analysis to quantify the relative transcription profile of key components of all stages of the autophagy process. Interestingly, *atg*8a was induced by 24 hpi and reduced back to its basal level towards the end of lytic infection (Fig. 2c). Conversely, *atg*8b was not induced upon infection, but is probably present during infection (identified by multiple lapidated forms of Atg8 in the western analysis; Fig. S3b). These results suggest that Atg8a and Atg8b play different roles during infection of *E. huxleyi*. While we do not know what the role of Atg8b is, we suggest that Atg8a, up-regulated in the later stages of infection, is responsible for the majority of the Atg8 activity needed for the lytic phase of infection and assembly. Selectivity of the autophagy process is achieved in part by the differential binding of the cargo or intermediate proteins such as p62 to Atg8 (Noda *et al*., 2010; von Muhlinen *et al*., 2013). When comparing the protein sequence of the two *E. huxleyi* Atg8 proteins, we found differences in the sequences of the binding sites to the cargo proteins, such as the EXXXI and the IPVIC residues at the C terminus of the protein (Fig. S3a). These sequence modifications could lead to a difference in selectivity of the two proteins, and reinforce the idea that they have distinctive functions within the cell. Lipidation of Atg8 by PE is a major indicator of activation of the autophgic processes and is considered a hallmark of active autophagy (Kirisako *et al*., 1999). Indeed, we observed a significant increase in lipidated Atg8 during infection (Fig. S3b). Treatment with phospholipase D (PLD) shifted the proteins back to their nonlipidated form, detected by the slower migration of the protein in the 6 M urea gel.

We further analyzed the transcript abundance of *vps*34, which is involved in the autophagy activation phase, and genes encoding for key proteins that are essential for autophagosome assembly and elongation (*atg*5 and *atg*7, Fig. 2c). We identified a homolog of *vps*34 in *E. huxleyi*, and showed that it is up-regulated during viral infection (Fig. 2c). Interestingly, in yeast and mammalian systems, the regulation of Vps34 is mainly on the protein level (Kim *et al*., 2013). This could explain the late transcription response of *vps*34 in infected *E. huxleyi*.

Both Atg5 and Atg7, which are essential for Atg8 lipidation and elongation of the autophagosome, are highly induced by 24 hpi (Fig. 2c). This suggests that there is an active positive regulation of the autophagy-like process throughout infection.

### The autophagy-like cellular process is essential for viral release

In order to get a direct link for the involvement of autophagy in mediating viral replication, we followed the course of infection after application of wortmannin (Fig. 3), an inhibitor of the critical activator of autophagy, PI3K (Codogno & Meijer, 2005). PI3K is encoded by *vps*34 and is induced during lytic infection (Fig. 2c). When *E. huxleyi* cells were preincubated with 1 μM wortmannin before viral infection, a significant decrease of *c*. 70% in viral yield was observed (Fig. 3b) without inhibiting viral-induced cell death. This profound reduction in viral release by inhibiting the autophagy-like process was not the result of reduced viral DNA replication, or of inhibition of transcription of viral genes (Figs 3a, S4). In the wortmannin-treated infected cells, we observed swollen endosome-like structures that accumulated viruses within them (Fig. 3d,f,h); these structures were not observed in the control cells or in the nontreated infected cells, where the autophagy-like process was not inhibited (Fig. 3c,e,g). The viruses were mostly aggregated within these swollen compartments and some of them appear to be budding into the internal endosome-like lumen. Interestingly, wortmannin has been shown to induce swelling of endocytic vacuoles in mammalian cells. This treatment did not affect the recruitment of membranes into the vacuole, but rather inhibited the export of membranes outward to other cellular destinations, resulting in the swollen phenotype (Bright *et al*., 2001). In the case of EhV, it is therefore tempting to speculate that viral egress is facilitated by a similarly polarized process. Inhibition of this process by wortmannin would lead to swelling of the compartments as seen in Fig. 3(e). Interestingly, the viral genome encodes for a SNARE protein that is suggested to be involved in membrane trafficking (Wilson *et al*., 2005). This protein might serve as a vital regulator of trafficking for the egress of the viruses. Intriguingly, the electron-dense DNA core of the viruses (Figs 1f, 3e,g) was not observed in the wortmannin-treated samples (Fig. 3f,h), suggesting that the packaging of the viruses is defective. Surprisingly, there was no significant difference between the infectivity of viruses isolated from media of wortmannin-treated or nontreated cultures. At the same viral titer, extracellular viruses from the wortmannin-treated and nontreated cultures had infectivity values of 24.2 ± 5.4 and 26.2 ± 5.6 plaque-forming units per viral particle, respectively (average ± SE, *P* = 0.8, *n* = 9, see the Materials and Methods section). This indicates that the inhibition of autophagy was not enough to completely inhibit production of infective virions.

**Figure 4:**
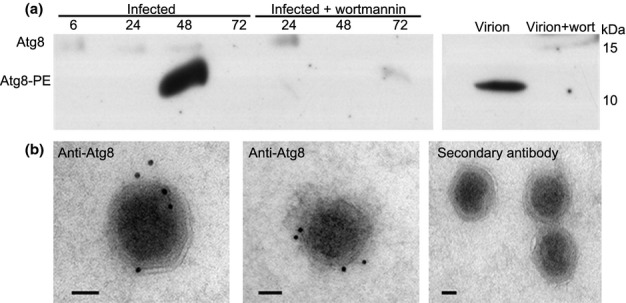
*Emiliania huxleyi* Atg8-PE accumulates in infected cells and in purified virions. (a) Western blot analysis using the anti-Atg8 antibody against protein extracts from infected *E. huxleyi* cultures (in RIPA lysis buffer) with or without 1 μM wortmannin throughout the course of infection. Purified virions from infected cultures and from cultures infected in the presence of wortmannin are also shown (two right lanes). (b) Immunotransmission electron microscopy (immuno-TEM) analysis was performed by hybridizing an anti-Atg8 antibody with purified virions using the Tokuyasu method (Tokuyasu, 1986). Seventy per cent of the 37 virions observed were positively cross-reacted with the Atg8 antibody. In the right-hand image, only the anti-rabbit secondary antibody was used as a negative control; none of the 35 virions observed were labeled by this treatment. Bars, 50 nm.

Western blot analysis of the Atg8 protein during infection revealed that the lipidated Atg8-PE is the predominant form in cells during infection, but only trace amounts of the protein were detected when pretreating infected cells with the inhibitor (Fig. 4a). This suggests that wortmannin blocks accumulation of Atg8 during infection, supporting its role in inhibition of the autophagy-like process in infected *E. huxleyi* cells. Intriguingly, cross-hybridization of the Atg8 antibody by western analysis revealed the host-encoded Atg8-PE form in protein extracts from purified virions (Fig. 4a). We could only detect residual amounts of the nonlipidated Atg8 in protein extracts of virions isolated from an infected culture that was treated with wortmannin. The presence of host Atg8-PE in the virions was confirmed by immuno-TEM analysis, whereby 70% (26/37) of the observed virions cross-reacted with the Atg8 antibody. In the negative control, where the virions were reacted with only the secondary antibody, none of the 35 virions observed were positive (Fig. 4b). Recent lipidomic studies further corroborated these findings by providing compelling evidence for intracellular induction of PE during infection of *E. huxleyi* by EhV, as well as detection of PE in the virion lipidome, where it represented 1.5% of the total lipids (Fulton *et al*., 2014). These data show that the membranes originating from the autophagic-like process within the infected cells are incorporated into the newly formed viral structures before egress from the cells. This is the first demonstration of incorporation of cellular autophagy components into viral structures, and it exemplifies the major role that cellular processes such as autophagy play in viral replication.

The results presented here suggest a novel mechanism of an NCLDV virus replication cycle, whereby induction of an autophagy-like process is essential for viral propagation. Moreover, we suggest that the Atg8-PE-containing DMVs are the building blocks of the internal double membrane structures of EhV. Interestingly, we could not detect Atg8-PE in isolated virions that were released from cells infected in the presence of wortmannin (Fig. 4a). This suggests that EhV requires the autophagic-like membrane for proper assembly, and that at least a fraction of its membranes originate from an Atg8-PE-containing membrane, most likely the autophagosome. Vaccinia virus, also a member of the NCLDV clade, contains large amounts of PE in the virion (Sodeik *et al*., 1993) and induced lipidation of the mammalian homolog of Atg8 (LC3) by an Atg5/Atg7 independent process, concomitant with inhibition of innate cellular autophagy (Moloughney *et al*., 2011).

The interplay between autophagy and cell death is well documented in mammalian systems (Young *et al*., 2013). Atg5, which was strongly up-regulated during EhV infection (Fig. 2c), was recently shown to serve as a switch between these two cellular processes (Yousefi *et al*., 2006; Zalckvar *et al*., 2010). It is tempting to speculate that these analogous mechanisms can both take place in *E. huxleyi* during late infection phases. It will be interesting to further investigate the link between viral-induced autophagy and caspase-dependent PCD following infection of *E. huxleyi* with EhV. Depending on the type and severity of stress conditions, different sphingolipid species can signal a switch between autophagy and PCD in the cells (Young *et al*., 2013). During EhV infection, *E. huxleyi* accumulates viral glycosphingolipids that are essential constituents of virion membranes and act as important signaling lipids to induce host PCD in a dose-dependent manner (Vardi *et al*., 2009, 2012). We therefore suggest that accumulation of sphingolipids may have a role in induction of an autophagy-like process that would support the construction of the virions.

*Emiliania huxleyi* virus (EhV) is a large virus that possesses at least two internal membranes (Fig. 1f–h), the majority of which are viral-specific sphingolipids, encoded for by the viral unique genome (Wilson *et al*., 2005; Mackinder *et al*., 2009; Pagarete *et al*., 2009; Vardi *et al*., 2009, 2012). We propose that induction of cellular autophagy may serve as the mechanism for recycling membranes that are needed for building the virion structure. We suggest that during the host–virus arms race, the virus subverts the cellular autophagic-like process for its benefit. By doing so, EhV can maximize its viral yield. This will raise the multiplicity of infection within the bloom, and, with it, the contact rate, thus increasing the chances of propagating more efficiently through blooms of its specific host. Taken together, the results presented here clearly imply a pivotal role for an autophagic-like process in viral infection of the bloom forming *E. huxleyi*. Consequently, the cross-talk between autophagic, sphingolipid metabolism and cell death processes may have major impact on the fate of the viral-infected blooms, and therefore on the cycling of nutrients and C within the microbial food webs in the marine ecosystem.

## Supporting Information

**Fig. S1** *E. huxleyi* displayed MDC staining during infection. **Fig. S2** Inhibition of lysosomal acidification does not affect viral replication or release. **Fig. S3** The *E. huxleyi* Atg8 homologs are lipidated during infection. **Fig. S4** Expression of viral genes is not affected by wortmannin. **Fig. S5** TEM analysis of infected *E. huxleyi* cells (large version of Fig. 1c–e). **Fig. S6** *E. huxleyi* displays hallmarks of an autophagy-like process during infection (green-magenta version of Fig. 2a). **Table S1** Primers used in this study
