# Supplementary material for: Hijacking of an autophagy-like process is critical for the life cycle of a DNA virus infecting oceanic algal blooms
Source: New Phytol. 2014 Sep 7;204(4):854–63. doi: 10.1111/nph.13008 (PMC4233938; doi:10.1111/nph.13008)
Supplement: Supplementary file 1 — Fig. S1 E. huxleyi displayed MDC staining during infection. Fig. S2 Inhibition of lysosomal acidification does not affect viral replication or release. Fig. S3 The E. huxleyi Atg8 homologs are lipidated during infection. Fig. S4 Expression of viral genes is not affected by wortmannin. Fig. S5 TEM analysis of infected E. huxleyi cells (large version of Fig. 1c–e). Fig. S6 E. huxleyi displays hallmarks of an autophagy-like process during infection (green-magenta version of Fig. 2a). Table S1 Primers used in this study [file nph0204-0854-sd1.docx]

## *New Phytologist* Supporting Information Table S1 and Figs S1–S6

**Article title:** Hijacking of an autophagy-like process is critical for the life cycle of a DNA virus infecting oceanic algal blooms

**Authors:** Daniella Schatz, Adva Shemi, Shilo Rosenwasser, Helena Sabanay, Sharon G. Wolf, Shifra Ben-Dor and Assaf Vardi

The following Supporting Information is available for this article:

**Table S1** Primers used in this study

| **Gene** | **Primer name** | **Primer sequence** |
| --- | --- | --- |
| *atg8a* | Atg8a-F | cggccagtttgtgtacgtgat |
| *atg8a* | Atg8a-R | gcctgctcgggcttga |
| *atg8b* | Atg8b-F | cagcaggtcaacagccatca |
| *atg8b* | Atg8b-R | tcaaacgggtggtccttctt |
| *vps34* | vps34-F | tgcgtcgccatgtacttgat |
| *vps34* | vps34-R | gatatagccaaagtcgatgtggaa |
| *atg5* | Atg5CL-F | gcgagggtaggtccagca |
| *atg5* | Atg5CL-R | gagaagctactggtactgcggttt |
| *atg7* | Atg7CL1-F | ccacacgtcacacgtacctgta |
| *atg7* | Atg7CL1-R | ggtggcgtcgttatacgttaaaa |
| *tubulin** | Tub-FW | cgctgtacgacatctgctt |
| *tubulin** | Tub-RV | ggaaggggatcatgttgac |
| *spt** | EhV-spt-F | agtccggtatcgtcttgtcg |
| *spt** | EhV-spt-R | cgcaatgcgataatacatgg |
| *mcp** | mcp1Fw | acgcaccctcaatgtatggaagg |
| *mcp** | mcp90Rv | agccaactcagcagtcgttc |

*According to (Pagarete *et al.*, 2009)

**References**

**Pagarete A, Allen MJ, Wilson WH, Kimmance SA, de Vargas C. 2009.** Host-virus shift of the sphingolipid pathway along an *Emiliania huxleyi* bloom: survival of the fattest. *Environ. Microbiol.* **11**(11): 2840-2848.

**
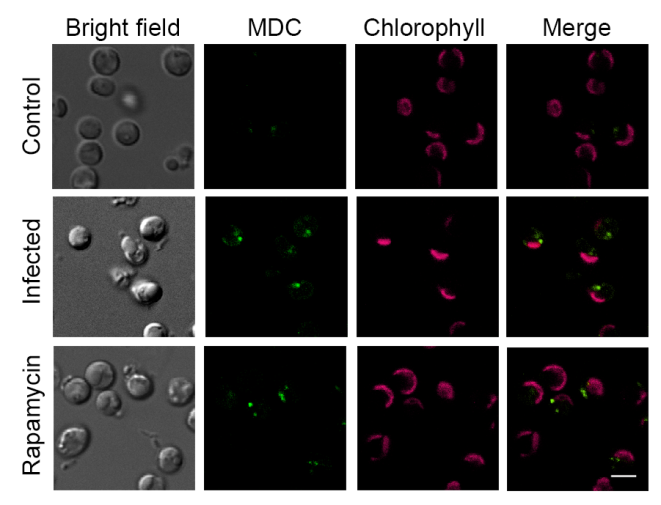
**

**Fig. S1** ***E. huxleyi* displayed MDC staining during infection.** Confocal micrographs of control, infected cells 24 h post infection (hpi), or cells treated with rapamycin (positive control) that were stained with MDC for detecting acidic compartments within the cells. Green, MDC stain; Magenta, chlorophyll autofluorescence. Bar, 3 µm.

**
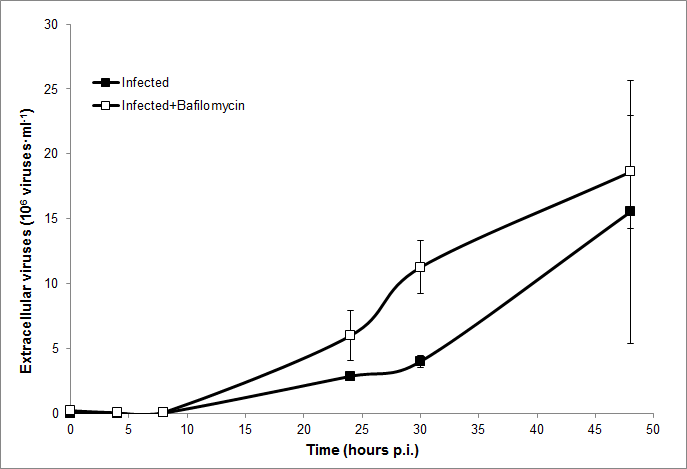
**

**Fig. S2 Inhibition of lysosomal acidification does not affect viral replication or release.** Assessment of host-virus dynamics in the presence of 50 nm bafilomycin by quantification of extracellular viral counts during infection with (closed squares) and without (open squares) 50 nM bafilomycin, estimated by qPCR using primers for the *mcp* gene (*n*=3, results presented are average ± SD).

**
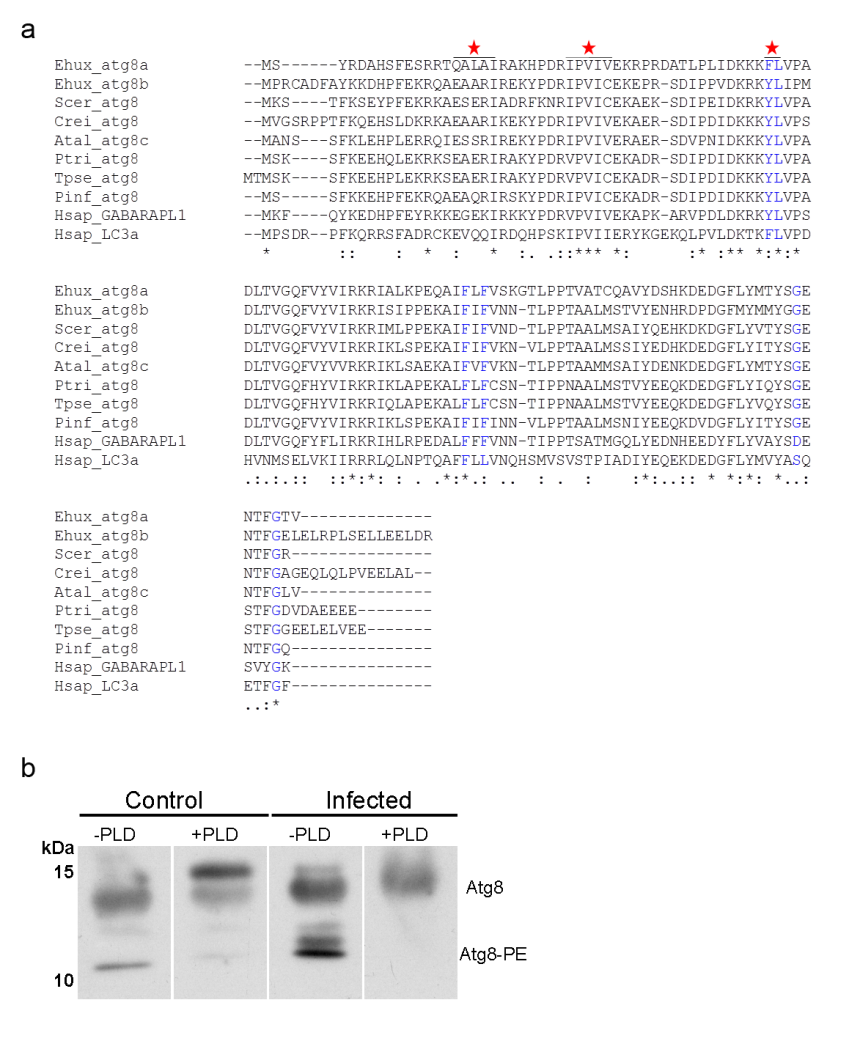
**

**Fig. S3 The *E. huxleyi* Atg8 homologs are lipidated during infection.** (a) Alignment of the two homologs of *E. huxleyi* Atg8 (Ehux_atg8a, Ehux_atg8b) with Atg8 proteins from the yeast *Saccharomyces cerevisiae* (Scer_Atg8), the green algae *Chlamydomonas reinhardtii* (Crei_Atg8), the plant *Arabidopsis thaliana* (Atal_Atg8c), the diatoms *Phaeodactylum tricornutum* (Ptri_Atg8) and *Thalassiosira pseudonana* (Tpse_atg8), the oomycete *Phytophthora infestans* (Pinf_atg8) and two homologs of the human Atg8 protein (Hsap_GABARAPL1 and Hsap_LC3a). Amino acids important for determining specificity of the protein are marked by an asterisk. Residues in blue are essential for Atg8 cleavage and lipidation. (b) Western analysis with an anti-Atg8 antibody against proteins extracted (in lysis buffer without detergent) from control or infected cultures 48 h post infection (hpi). Samples were treated with phospholipase D (PLD) to de-lipidate the proteins. The lipidated form of Atg8 is apparent by its faster migration within the gel.

**
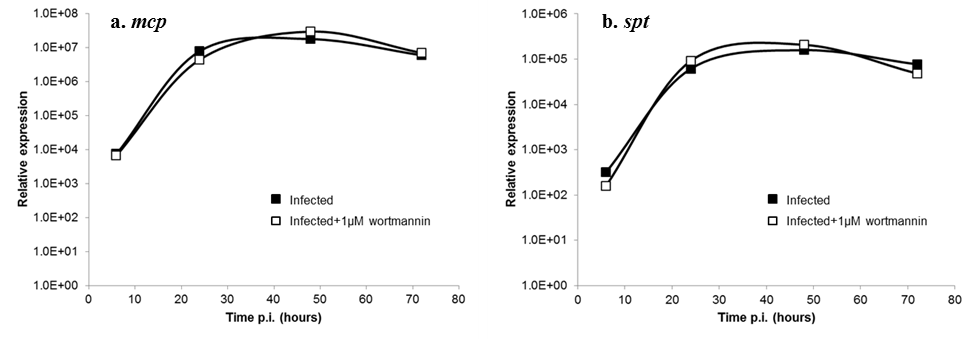
**

**Fig. S4 Expression of viral genes is not affected by wortmannin.** RT-PCR analysis of the virally encoded *spt* (a) and *mcp* (b) genes throughout infection with (open squares) or without (closed squares) 1 µM wortmannin.


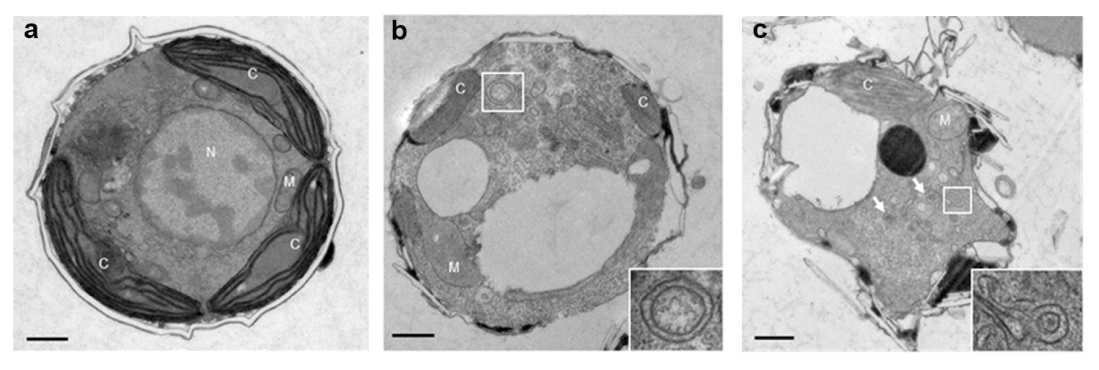


**Fig. S5** **TEM analysis if infected *E. huxleyi* cells.** (a) TEM analysis of a representative micrograph of *E. huxleyi* control cells. (b, c) Infected cells 24 hours post infection by EhV. Degradation of mitochondria (M) and chloroplasts (C) are apparent. Arrows point to DMVs. Bars, 500 nm. The insets in (b) and (c) show higher magnification of the boxed areas, depicting the DMVs. This figure is a large version of Fig 1(c–e).


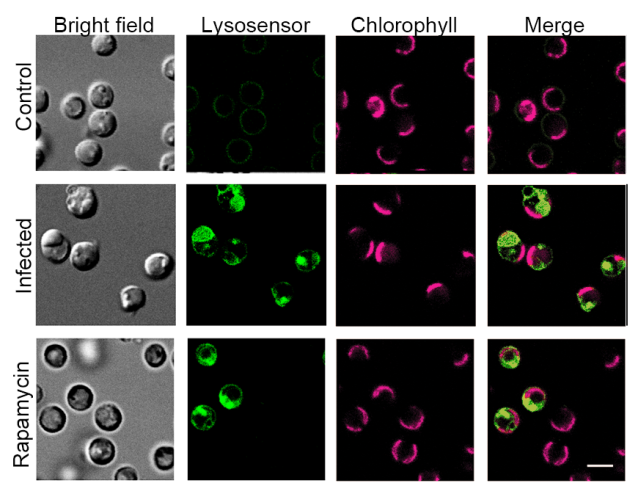


**Fig. S6 *E. huxleyi* displays hallmarks of an autophagy-like process during infection.** Confocal micrographs of control, infected cells 24 h post infection (hpi), or rapamycin treated cells (positive control) that were stained with Lysosensor for detecting acidic compartments within the cells. Green, Lysosensor stain; Magenta, chlorophyll autofluorescence. Bar, 3 µm. This image is identical to Fig. 2(a) but magenta was used instead of red.
